# Nanobody-based Pannexin1 channel inhibitors increase survival after cardiac ischemia/reperfusion

**DOI:** 10.1007/s00441-025-03994-y

**Published:** 2025-07-23

**Authors:** Olga M. Rusiecka, Filippo Molica, Linda Clochard, Raf Van Campenhout, Timo W. M. De Groof, Viviane Bes, Nick Devoogdt, Serge Muyldermans, Mathieu Vinken, Brenda R. Kwak

**Affiliations:** 1 Department of Pathology and Immunology, Faculty of Medicine, University of Geneva, Geneva, Switzerland; 2 Geneva Center for Inflammation Research, Faculty of Medicine, University of Geneva, Geneva, Switzerland; 3 Department of Pharmaceutical and Pharmacological Sciences, Faculty of Medicine and Pharmacy, Vrije Universiteit Brussel, Brussels, Belgium; 4 Molecular Imaging and Therapy Research Group, Faculty of Medicine and Pharmacy, Vrije Universiteit Brussel, Brussels, Belgium; 5 Laboratory of Cellular and Molecular Immunology, Faculty of Sciences and Bioengineering Sciences, Vrije Universiteit Brussel, Brussels, Belgium

**Keywords:** Pannexin1, Channels, Cardiac ischemia/reperfusion, Endothelial cells, Nanobodies

## Abstract

Reperfusion following myocardial infarction salvages the ischemic heart but paradoxically exacerbates injury. Yet, efficient treatment for cardiac ischemia/reperfusion injury is still missing in clinics. ATP release through Pannexin1 (PANX1) channels facilitates recruitment of leukocytes to the injured myocardium. Thus, PANX1 channel inhibition might confer cardioprotection. Currently available PANX1 channel blockers lack specificity or *in vivo* stability. Nanobodies offer a new therapeutic modality given their high target affinity, small size, and deep tissue penetration. Nanobodies targeting Panx1 were recently introduced. Here, their target specificity and selective PANX1 channel inhibition for cardiovascular purposes were validated in vitro. The two most promising candidates were further examined in the context of cardiac ischemia/reperfusion injury. Nanobody-1 (Nb1) and Nb9 reduced neutrophil adhesion to an endothelial monolayer. Nb1 did not affect left ventricular function ex vivo; however, Nb9 tended to diminish the performance of isolated hearts. Finally, *in vivo* application of Nb1, but not of Nb9 or a control Nb, at the onset of reperfusion increased the survival rate of mice. However, the infarct size observed after treatment with Nb1 was similar than the one found after treatment with the control Nb. In conclusion, Nb1 efficiently and specifically inhibits ATP release from endothelial cells thereby limiting leukocyte adhesion and improving the outcome of cardiac ischemia/reperfusion in mice. This warrants further studies to unveil the detailed molecular mechanism underlying the beneficial effects of Nb1.

## Introduction

Ischemic heart disease accounts worldwide for nearly 9 million deaths each year (Timmis, et al., 2022). Myocardial ischemia and infarction occur when blood flow to the myocardium is restricted, often as a result of erosion or rupture of an atherosclerotic lesion resulting in thrombus formation and occlusion of a coronary artery (Anderson and Morrow 2017). Treatment consists of procedures that allow the rapid return of blood flow to the ischemic zone to rescue heart muscle. Although it is essential to re-open the occluded coronary artery as early as possible, reperfusion paradoxically leads itself to further complications involving acceleration of cardiomyocyte death, diminished contractile function, and heart failure in the long term (Heusch 2020). Thus, additional cardioprotective measures are needed on top of timely reperfusion.

The cardiac damage after ischemia/reperfusion (I/R) results from a delicate balance between cardiomyocyte death, inflammation, and tissue repair. Effective cardioprotective therapy is therefore expected to involve multiple cell targets, including the prevention of an exaggerated inflammatory response (Davidson, et al. 2019). Pannexin1 (PANX1) channel activation has been associated with the onset and progression of many inflammatory pathological conditions (Crespo Yanguas, et al. 2017; Koval, et al. 2021; Martins-Marques, et al. 2025; Rusiecka, et al. 2022). PANX1 is a ubiquitously expressed glycoprotein (Boassa, et al. 2007, 2008; Penuela, et al. 2007, 2009) that forms heptameric membrane channels in the plasma membrane (Deng, et al. 2020; Michalski, et al. 2020; Qu, et al. 2020) allowing for the controlled release of adenosine 5′-triphosphate (ATP) (Chiu, et al. 2018; Dahl 2018; Gupta, et al. 2025; Ruan, et al. 2020). PANX1 channels are predominantly closed under physiological conditions, and their open probability increases in response to various pathological stimuli such as oxygen/glucose deprivation, elevated extracellular K^+^, high intracellular Ca^2+^, and mechanical stretch (Chiu, et al. 2018). Indeed, ATP release through PANX1 channels has been shown to regulate NLRP3 (NOD-, LRR-, and pyrin domain-containing protein 3) inflammasome activation (Parzych, et al. 2017), to contribute to leukocyte activation and chemoattraction to inflamed or injured areas (Good, et al. 2021; Lohman, et al. 2015) and to facilitate a positive feedback loop in T lymphocytes, leading to their sustained activation (Woehrle, et al. 2010). Using (cell-specific) Panx1-deficient mouse models, several studies have demonstrated that PANX1 channels regulate various diseases characterized by an exacerbated inflammatory response, including atherosclerosis (Molica, et al. 2017), stroke (Freitas-Andrade, et al. 2017), and cardiac and renal ischemia/reperfusion injury (Good, et al. 2021; Jankowski, et al. 2018; Rusiecka, et al. 2023).

In an *in vivo* model of stroke, pharmacological inhibition of PANX1 channels with the clinically used uricosuric drug probenecid decreased infarct size and reduced inflammatory cell recruitment (Freitas-Andrade, et al. 2017). Similar outcomes were obtained with probenecid during I/R of the heart, lung, and kidney (El-Maadawy, et al. 2022; Good, et al. 2021; Sharma, et al. 2018). Interestingly, in a large cohort study of elderly patients with gouty arthritis, probenecid treatment was associated with a lower risk of cardiovascular events, including myocardial infarction, than treatment with the alternative uric acid–lowering drug allopurinol (Kim, et al. 2018). Finally, a relatively selective mode of PANX1 channel inhibition was attributed to the FDA-approved food dye Brilliant Blue-FCF (Wang, et al. 2013). PANX1 channel blockade with Brilliant Blue-FCF has been shown to reduce collagen-induced platelet aggregation in vitro and in vivo and prompted us to develop a more selective mini-antibody–based approach to inhibit thrombosis in mice (Molica, et al. 2019).

Currently, nanobodies (Nbs) are considered interesting alternatives to monoclonal antibodies and other drug types to block ion channels and receptors (Blanchetot, et al. 2013, Danquah, et al., 2016, McMahon, et al. 2020). Thanks to the lack of the light chain in their structure, nanobodies display better extravasation and tissue penetration than conventional antibodies and can target cryptic epitopes such as binding pockets and clefts (Hassanzadeh-Ghassabeh, et al. 2013; Jovcevska and Muyldermans 2020; Van Campenhout et al. 2023). Moreover, the high plasma stability of nanobodies and the lack of immunogenic reaction after their administration support their use as therapeutics (Muyldermans 2021).

Recently, PANX1-targeting nanobodies have been introduced (Van Campenhout et al. 2023). In the present study, the binding potential and cytotoxic effects of a number of these PANX1-targeting nanobodies have been tested using in vitro assays. We confirmed that these nanobodies specifically target and block PANX1 channels. Subsequently, the two best nanobody candidates were selected to validate their cardioprotective potential in a mouse model of cardiac *I*/*R*. We successfully demonstrate that one of these PANX1-targeting nanobodies improves the survival of mice subjected to myocardial I/R without disturbing physiological cardiac function.

## Materials and methods

### Animals

All experimental protocols involving mice were reviewed and approved by the Swiss cantonal and federal veterinary authorities (number 33646). All animal experiments were performed in accordance with relevant guidelines and regulations, and the reporting in this study is in accordance with the ARRIVE guidelines 2.0 published by the National Centre for the Replacement, Refinement & Reduction of Animals in Research (NC3R^s^). All mice were kept in a conventional animal facility under controlled environmental conditions (room temperature 20–24 °C, humidity 30–70%, 12/12 h light/dark cycle) with free access to food and water. The cages were enriched with bedding, nestlet, and mouse house.

### Production of nanobodies

The production of anti-PANX1 nanobodies is described elsewhere in detail (Van Campenhout et al. 2023). To verify the quality of the final product, the nanobodies were purified on an AKTAxpress chromatography system equipped with a HiLoad S75 (16/60) column and treated for endotoxin removal. The nanobodies were prepared in 0.9% NaCl at a concentration of 3 mg/mL. Seven Nbs against PANX1, namely, Nb1, Nb3, Nb6, Nb9, Nb16, Nb20, and Nb30, have been tested in vitro in endothelial cells and cardiac myoblasts, devoid of PANX1, for their specificity and for potential cytotoxicity using ATP release and LDH assays, respectively. For further ex vivo and in vivo experiments, a non-targeting control nanobody (NbR3b23 (Lemaire, et al. 2014)) was used.

### Cell culture

#### Dubai camel fibroblasts (DUBCA)

Parental DUBCA cells and DUBCA cells transduced with a mouse Panx1 construct (DUBCA^mPanx1^) (Van Campenhout et al. 2023) were grown in modified Eagle medium (MEM, Gibco) supplemented with 10% fetal bovine serum (FBS) and 1% antibiotics (penicillin–streptomycin, P/S) at 37 °C and 5% CO_2_. To maintain the culture, cells were passaged every 2 days after trypsinization (0.05% Trypsin–EDTA, Gibco; 2 min; 37 °C) followed by centrifugation (1200 rpm; room temperature (RT); 5 min) and plated (1:3 dilution).

#### Chinese hamster ovary cells (CHO)

CHO cells were grown in RPMI 1640 medium supplemented with GlutaMAX (Gibco) containing 10% FBS and 1% P/S at 37 °C and 5% CO_2_. Cultures were maintained by passaging the cells every 2 days after trypsinization (0.05% Trypsin–EDTA, Gibco; 2 min; 37 °C). Cells were transfected with an empty vector (CHO^EV^) or with a human Panx1-GFP containing plasmid (CHO^hPanx1−GFP^) (Molica, et al. 2015) during 32 h prior to lysis for protein extraction.

#### Human vascular endothelial cells (EA.hy926)

The human umbilical vein cell line EA.hy926 (ATCC no CRL-2922) was grown in Dulbecco’s modified Eagle medium (DMEM, Gibco) supplemented with 10% FBS and 1% P/S at 37 °C and 5% CO_2_. The cell culture was maintained by passaging every 2 days by incubation with Trypsin–EDTA (2 min at 37 °C) and centrifugation (1200 rpm; RT; 5 min). Pellets were resuspended and plated in culture dishes pre-coated with 1.5% gelatin.

#### Rat cardiomyoblast cells (H9c2)

Rat cardiac myoblasts (H9c2; ATCC no CRL-1446) were grown in DMEM supplemented with 10% FBS and 1% P/S (37 °C, 5% CO_2_) and passaged every 2 days by trypsinization (2 min, 37 °C) followed by centrifugation (1200 rpm; RT; 5 min). Cells were differentiated as described elsewhere (Branco, et al. 2015).

#### Human myeloid cells (PLB-985)

PLB-985 cells (kindly provided by Prof. Karl-Heinz Krause, University of Geneva, Switzerland) (Seredenina, et al. 2015) were grown in T-25 flasks in Roswell Park Memorial Institute medium (RPMI)−1640 (Gibco) medium supplemented with 10% FBS and 1% P/S. Differentiation into mature neutrophils was performed for 3, 5 and 7 days (37 °C, 5% CO_2_) by the addition of 1.25% DMSO to the cell culture medium.

### Flow cytometry analysis

EA.hy926 or H9c2 cells were collected, rinsed with ice-cold FACS buffer (PBS with 1% bovine serum albumin), plated into 96-well plates, and incubated with nanobodies at a concentration of 100 nM or 300 nM for 1.5 h at 4 °C. Cells were then washed three times with FACS buffer by centrifugation (1400 rpm, 2.5 min, 4 °C), and incubated with a secondary antibody against Hemagglutinin tag (1/100; Biolegend) for 30 min at 4 °C. Panx1 expression was also detected using the anti-PANX1 HRB454 mini-antibody (Geneva Antibody Facility, University of Geneva) (Molica, et al. 2019). Briefly, after washing, the cells were fixed in 4% paraformaldehyde (PFA) for 15 min, blocked in 2% BSA for 15 min, and stained overnight with HRB454 (1/250) in FACS buffer at 4 °C. Incubation with secondary anti-human antibody (1/1000; ThermoFisher Scientific) was performed for 2 h at 4 °C. Cells were washed three times (1400 rpm, 2.5 min, 4 °C) and were counterstained with 4′,6-diamidino-2-phenylindole (DAPI) to assess their viability. Finally, cells were resuspended in FACS buffer, and Panx1 expression was analyzed by flow cytometry (Beckman Coulter Cytoflex). The affinity of Nb1 and Nb9 was evaluated by incubating EA.hy926 cells with the Nbs at different concentrations ranging from 0 to 600 nM in three independent experiments. Recorded binding values were used to define equilibrium dissociation constant (*K*_*d*_) values using previous methods (Van Campenhout et al. 2023).

### Western blotting

Proteins were extracted from DUBCA, DUBCA^mPanx1^, CHO^EV^, and CHO^hPanx1−GFP^ cells using a RIPA lysis buffer (1% NP40; 30 mM NaCl; 50 mM Tris; 10 mM NaF; 2 mM Na_3_VO_4_; 1 mM PMSF; 1 mM EDTA; 0.05% SDS; 0.2% Na-deoxycholate; supplemented with a proteases inhibitor cocktail). Briefly, cells were rinsed with ice-cold PBS, centrifuged (1200 rpm; 20 min; 4 °C), resuspended in RIPA buffer, and incubated on ice for 20 min while gently mixing every 5 min. Samples were centrifuged (14,000 rpm; 20 min; 4 °C), and supernatants were collected. Protein concentration was assessed using a copper-based total Micro BCA protein assay quantification kit (ThermoFisher Scientific) according to the manufacturer’s instructions. PANX1 expression was verified by Western blot using a rabbit anti-Panx1 primary antibody (1/1000; Cell Signaling, cat. no. 91137) and the corresponding secondary anti-rabbit antibody (1/5000; Jackson Laboratories). Chemiluminescent signal was detected using the Immobilon ECL Ultra Western HRP Substrate (LAS 2000, Millipore).

### Real-time quantitative polymerase chain reaction

mRNA was extracted from EA.hy926, H9c2 and PLB-985 cell lines and tissues with the NucleoSpin RNA Mini kit (Macherey–Nagel), and reverse transcription was performed using the Quantitect Reverse Transcription kit (Qiagen). As a positive control and respecting 3R regulations, kidney and liver samples were obtained from control Wistar rat cadavers within 10 min after their sacrifice for another study (authorization number GE153). Real-time PCR was performed with the ABI Prism StepOnePlus Sequence Detection System (Applied Biosystem) using primers for human Panx1 (cat. no. Hs00200790-m1) and glyceraldehyde-3-phosphate dehydrogenase (gapdh; cat. no. Hs02758991-g1), vascular cell adhesion molecule-1 (vcam-1; cat. no. Hs01003372-m1), and intercellular adhesion molecule-1 (icam-1; cat no. Hs00164932-m1). Primers for rat Panx1 (cat. no. Rn01447976-m1) and rat gapdh (cat. no. Rn01775763-g1) were used for H9c2 cells. All primers were purchased at Applied Biosystems. Gene expression was normalized to corresponding gapdh.

### Immunofluorescence staining

DUBCA and DUBCA^mPanx1^ cells were stained with anti-PANX1 HRB462 mini-antibody (1/250; Geneva Antibody Facility) following a previously described protocol (Rusiecka, 2019). Briefly, following fixation with 4% paraformaldehyde (PFA, 15 min), cells were permeabilized in 0.3% Triton X-100 (15 min), incubated in 0.5 M NH_4_Cl (15 min), and then blocked with 2% bovine serum albumin solution (45 min). Staining was performed overnight at 4 °C. Panx1 signal visualization was carried out with a secondary goat anti-human (1/2000, Jackson Laboratories) antibody. Cells were counterstained with 0.003% Evans blue, nuclei were stained with DAPI (10 min, 1/20,000), and the slides were mounted in Vectashield Antifade Mounting Medium (VECTOR Laboratories). Images were captured using a Zeiss Axio Imager Z1 with an EC Plan-Neofluar 40x/1.3 Oil (42,462.9900) objective.

### LDH activity assay

Potential cytotoxic effects of the Nbs were assessed on cell supernatants of EA.hy926 and H9c2 cells using a lactate dehydrogenase (LDH) activity assay (Sigma-Aldrich, cat. no. 11644793001) according to the manufacturer’s instructions. As a positive control, cells were lysed using 2% of the detergent Triton X-100. Absorbance was measured at 450 nm with Ledetect96 Microplate Reader (Bioconcept).

### ATP release assay

EA.hy926 and H9c2 cells were plated on 96-well plates pre-coated with 1.5% gelatin (EA.hy926) or non-coated (H9c2) and grown to confluency. Cells were rinsed with PBS and incubated in Tyrode buffer (pH = 7.4; Osm = 285 mΩ/kg) for 5 min at 37 °C. Cells were then incubated with ^10^Panx peptides (400 μM; Tocris, cat. no. 3348) or with nanobodies (each at 100 nM) diluted in Tyrode buffer (124 mM NaCl, 2.44 mM KCl, 10.82 mM NaHCO_3_, 0.38 mM NaH_2_PO_4_, 0.91 mM MgCl_2_, 1.82 mM CaCl_2_; pH 7.35; 295 mΩ/kg) for 10 min at 37 °C. PANX1 channel opening was induced by a hypo-osmotic shock (HOS) solution (30.24 mM NaCl, 10 mM KCl, 10.82 mM NaHCO_3_, 0.38 mM NaH_2_PO_4_, 0.91 mM MgCl_2_, 1.82 mM CaCl_2_; pH = 7.4; Osm = 136 mΩ/kg) in the presence of ^10^Panx peptides (400 μM) or nanobodies, and supernatants were collected after 30 min. ATP release was measured in supernatants with an ATP Bioluminescence Assay kit (Sigma-Aldrich, cat. no. FL-AAM-1VL) according to the manufacturer’s instructions with Lumat LB 9507 (EG&G Berthold).

### Adhesion assay

EA.hy926 cells (150,000/well) were plated in 24-well plates and cultured for 72 h to obtain a confluent monolayer. Cells were activated for 24 h with human tumor necrosis factor α (hTNFα; R&D Biosystems) at a concentration of 10 ng/mL (37 °C, 5% CO_2_). Vcam-1 and icam-1 expression was measured by quantitative RT-PCR to verify correct stimulation with TNFα. PLB-985 cells were differentiated into neutrophils using 1.25% dimethyl sulfoxide (DMSO) for 3 days. PLB-985 cells were stained with 5 μM 5–6-carboxyfluorescein diacetate, succinimidyl ester (CFDA-SE; ThermoFisher Scientific) as previously described (Wong, et al. 2006). Prior to the adhesion assay, both cell lines were incubated for 10 min with Nb1 or Nb9 (100 nM) or an equivalent milliliter of vehicle (0.9% NaCl) in RPMI-1640 medium. Probenecid (2.5 mM; Invitrogen, cat. no. P36400) was used as a positive control. The suspension of fluorescent-labeled PLB-985 cells (60,000 cells/well) was then added to the EA.hy926 cell monolayers and left to adhere for 3 h (37 °C, 5% CO_2_). Photographs were captured using a ZOE Fluorescent Cell Imager (Biorad). Adhered neutrophils were quantified by a blinded observer based on the green fluorescent signal of four chosen regions of interest (ROIs) within each well and expressed as a percent of the control condition (TNFα-stimulated cells). Non-activated EA.hy926 cells were used as a control for basal adhesion.

### *Ex vivo *Langendorff perfusion

C57BL/6 J (WT) male mice were anesthetized with an intraperitoneal (*i.p.*) injection of ketamine-xylazine (Graeub and Bayer, 120 and 16 mg/kg, respectively). When the mice were fully anesthetized, hearts were excised and canulated via the aorta to a Langendorff perfusion system. Mice were immediately thereafter euthanized by cervical dislocation and bloodletting. Isolated hearts were retrogradely perfused under constant pressure (approx. 70 mmHg) with gassed (94% O_2_, 6% CO_2_) Krebs–Henseleit buffer solution (118 mM NaCl, 4.7 mM KCl, 1.19 mM MgSO_4_, 1.2 mM KH_2_PO_4_, 1.36 mM CaCl_2_, 25 mM NaHCO_3_, 11 mM Glucose) at 37 °C, as described previously (Morel, et al. 2014, 2016, 2012). The left atrium was excised to expose the left ventricle entry to insert a silicon balloon connected to a pressure transducer to monitor the cardiac function (LabChart, ADInstruments). The heart was submerged into a warming chamber and kept at 37 °C throughout the procedure. Temperature control was ascertained with a temperature probe fixed next to the cannula. After 15 min of stabilization, Nb1, Nb9, or NbR3b23 (final concentration 100 nM) was added to the perfusate for 10 min. Recording was performed for up to 45 min. Analysis of the left ventricular function was performed offline with the LabChart software (ADInstruments) by a blinded observer.

### In vivo ischemia/reperfusion injury

WT male mice were injected subcutaneously with buprenorphine HCl (0.05 mg/kg in 100 μL) before the surgery, and anesthesia was induced with 4% isoflurane (Piramal Pharma) and confirmed with the absence of pedal reflex. Mice were then intubated via tracheotomy to allow for mechanical ventilation (tidal volume 150 μL; ventilation rate 120 breaths/minute; rodent respirator, Rothacher Medical GmbH). Anesthesia was maintained during the surgical procedure with 2% isoflurane and 100% O_2_ administrated through the ventilator. The heart was exposed by left-side thoracotomy followed by an incision between the third and the fourth intercostal space. A 30-min ischemia period was performed by ligation of the left anterior descending (LAD) coronary artery with a prolene suture 8–0 wound around a small piece of polyethylene catheter. Five minutes prior to reperfusion, Nb1, Nb9, or control NbR3b23 (20 mg/kg body weight; (Sadeghi, et al. 2020)) was injected via the tail vein. The snare was then released, the chest was closed with sutures, and the remaining air was evacuated from the chest cavity with a syringe. Animals were extubated and monitored for the following 24 h. After this time, animals were anesthetized with an *i.p.* injection of ketamine-xylazine (120 and 16 mg/kg, respectively). The chest of the mice was opened, and the LAD was re-occluded. Two percent Evans blue was immediately administered retro-orbitally. The hearts were immediately excised and rinsed with 0.9% NaCl before freezing at − 20 °C. Mice were immediately thereafter euthanized by cervical dislocation and bloodletting. Frozen hearts were sliced into 1-mm-thick transverse sections and stained with 0.25% triphenyltetrazolium chloride (TTC; pH = 7.4) for 40 min at 37 °C, which marked necrotic tissue in white allowing for discrimination of the infarcted area (IA). Specimens were post-fixed in 4% formol for 24 h and imaged with a digital camera (Nikon 1 J5 Model). The area at risk (AAR; as % of LV area) and infarct area (IA; as % of AAR) were quantified by a blinded observer using the ImageJ software.

### Statistical analysis

Statistical analysis was performed using GraphPad Prism 9. Comparisons were performed using a Student’s *t* test or one-way analysis of variance (ANOVA) where appropriate. The survival rate of animals subjected to *in vivo* I/R was analyzed with a contingency table using a Fisher’s exact test. Results were reported as mean ± SEM. Significant statistical results are indicated as *P ≤ 0.05, **P ≤ 0.01, ***P ≤ 0.001, or ***P ≤ 0.0001.

## Results

### Production of anti-Panx1 nanobodies

**Fig. 1:**
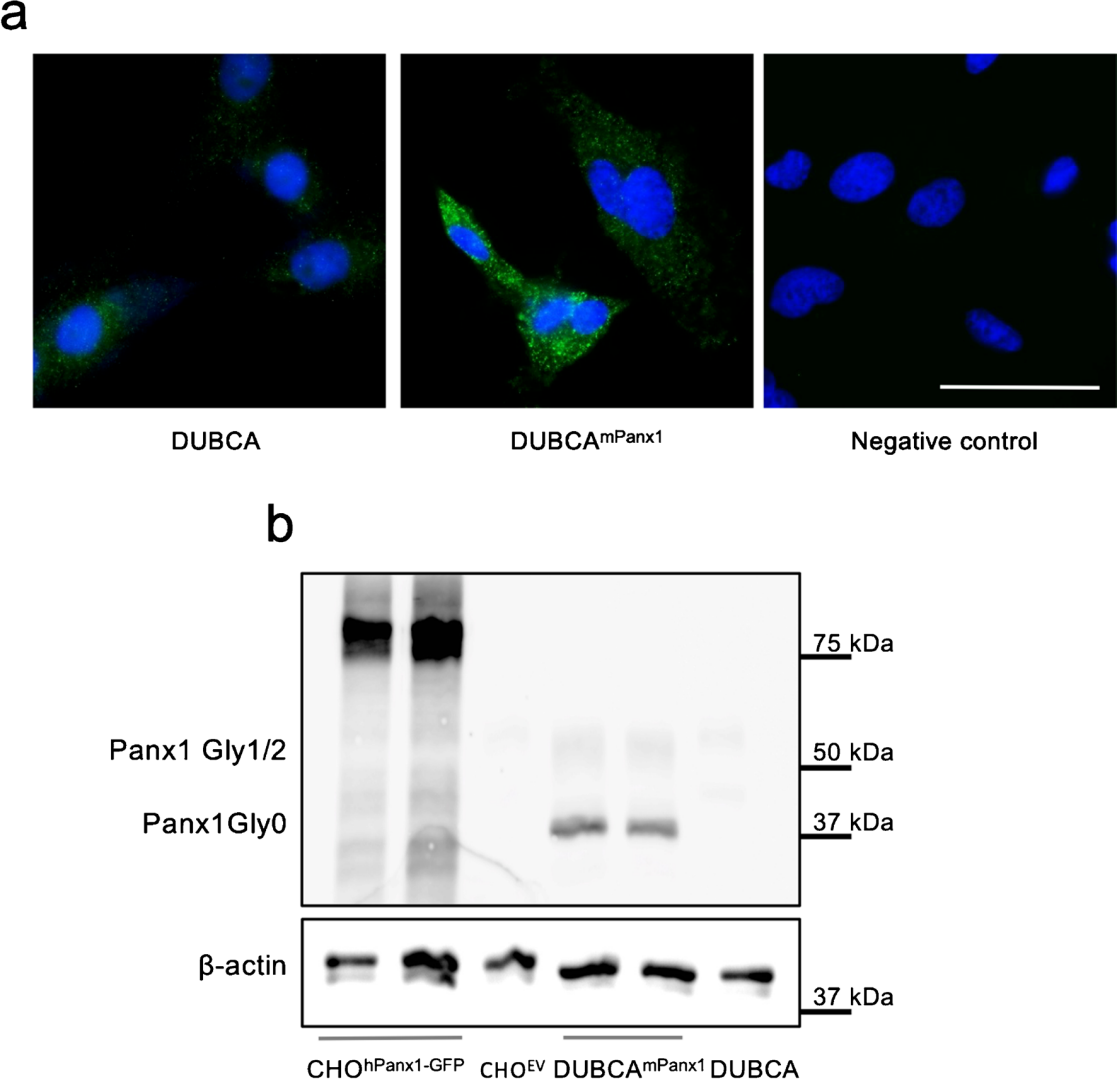
PANX1 expression in Dubai camel fibroblasts transfected with mouse Panx1 (DUBCA^mPanx1^). **a** PANX1 expression and cellular localization were confirmed by immunofluorescence. PANX1 was found in the cell membrane and in the cytosol of DUBCA^mPanx1^. The fluorescent signal was almost not detected in parental DUBCA cells and not present in the negative control (omission of primary antibody). DAPI (blue), scale bar = 50 μm. Representative photographs of three independent experiments. **b** Western blotting reveals bands for PANX1-Gly2 (48 kDa) and for PANX1 -Gly0/Gly1 (41–43 kDa) in DUBCA^mPanx1^ cells. These bands were virtually absent in parental DUBCA cells. Chinese hamster ovary (CHO) cells transfected with human Panx1-GFP (75 kDa) (CHO^hPanx1^) and empty vector-transfected cells (CHO^EV^) were used as a positive and negative control, respectively. β-actin (42 kDa) was used as a loading control. Representative image of two independent experiments

The production of PANX1-targeting nanobodies was carried out as recently described (Van Campenhout et al. 2023). Briefly, PANX1-targeting heavy chain-only antibodies were raised by immunization of a llama (*Lama glama*) with a mouse Panx1 (mPanx1) cDNA expression vector. The llama was subsequently boosted with a Dubai camel (DUBCA) fibroblast cell line transfected with mPanx1. Overexpression of PANX1 in DUBCA cells was confirmed by immunostaining (Fig. 1a). Whereas parental DUBCA cells showed a faint, mostly intracellular staining (left panel), PANX1 expression in DUBCA^mPanx1^ cells was located both intracellularly and at cell borders (middle panel). In addition, we detected by Western blotting multiple bands between 43 and 50 kDa in DUBCA^mPanx1^ cells (Fig. 1b), including the complex glycosylated (Gly2) plasma membrane–associated form of PANX1 (Penuela, et al. 2009). These bands were barely not detected in parental DUBCA cells and absent in Chinese Hamster Ovary (CHO) cells transfected with an empty vector (CHO^EV^ cells), which served as negative control. Of note, CHO cells transfected with human Panx1-GFP (CHO^hPanx1−GFP^) were used as positive control and showed indeed a band around 80 kDa as expected for the human PANX1-GFP fusion protein (Molica, et al. 2015).

Subsequently, cDNA from peripheral blood lymphocytes of the immunized llama was used to construct an immune nanobody library in pMECS-GG. Panx1-targeting nanobodies were retrieved using phage display and biopanning on the aforementioned DUBCA^mPanx1^ cells, resulting in the identification of seven different nanobodies. These nanobodies were further characterized for potential use to treat cardiac I/R in mice.

### Anti-PANX1 nanobodies bind to Panx1 channels in the membrane of endothelial cells

**Fig. 2:**
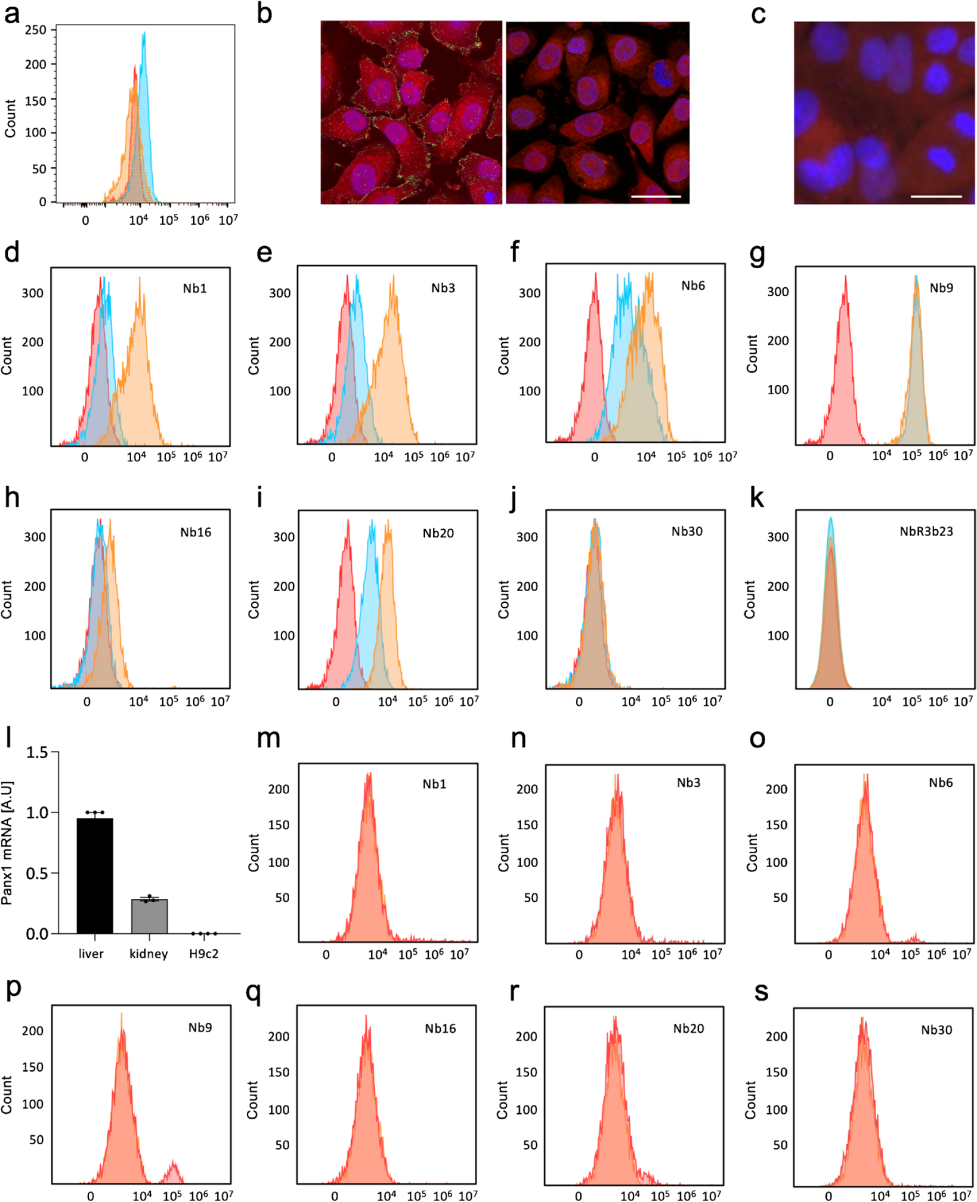
The specific binding of nanobodies Panx1 channels was verified by flow cytometry analysis. **a** Flow cytometry analysis shows PANX1 expression in EA.hy926 cells (blue) and its absence in H9c2 cells (orange). The average fluorescence intensity of EA.hy926 cells without primary antibody (red; negative control) was comparable to the H9c2 signal. Representative example of > 3 independent experiments. **b**, **c** Immunofluorescence staining confirms the presence of PANX1 (green, left panel) in **b** (left panel) EA.hy926 cells and **b** (right panel) absence of immunosignal from the negative control. **c** PANX1 is not detected in H9c2 cells. Evans blue (red), DAPI (blue), scale bar = 50 μm. Representative example of > 3 independent experiments. **d**–**k** EA.hy926 cells were stained with **d** Nb1, **e** Nb3, **f** Nb6, **g** Nb9, **h** Nb16, **i** Nb20, **j** Nb30, and **k** the control NbR3b23 at concentrations of 100 nM (blue) or 300 nM (orange). Flow cytometry analysis revealed a low background fluorescent signal in the negative control (red; omission of nanobody). Most nanobodies, except Nb30 and NbR3b23, bound to EA.hy926 cells albeit with different affinities. Representative examples of two independent experiments. **l** Quantitative RT-PCR analysis confirms the absence of Panx1 mRNA in H9c2 cells. Results are normalized to Panx1 mRNA expression in rat liver (black). Rat kidney samples were used as an additional positive control (grey). *N* = 3–4. **m**–**s** H9c2 were stained with **l** Nb1, **m** Nb3, **n** Nb6, **o** Nb9, **p** Nb16, **q** Nb20, and **r** Nb30 at a concentration of 300 nM (orange). Flow cytometry analysis revealed a low background fluorescent signal in the negative control (red; omission of nanobody). None of the nanobodies bound to Panx1-deficient H9c2 cells, illustrating the specificity of the nanobodies. Representative examples of two independent experiments

The panel of 7 PANX1-targeting nanobodies, namely, Nb1, Nb3, Nb6, Nb9, Nb16, Nb20, and Nb30 as well as a control nanobody (NbR3b23 (Lemaire, et al. 2014)), were tested for in vitro binding to endothelial cells using the human endothelial EA.hy926 cell line (Lamouroux, et al. 2023). The rat cardiomyocyte-like cell line H9c2 (Branco, et al. 2015), which lacks PANX1 expression, was used as a relevant (cardiac) control for the binding specificity of the PANX1-targeting nanobody panel. PANX1 expression at the plasma membrane of EA.hy926 cells and its absence on H9c2 cells were first confirmed by flow cytometry analysis using a mini-antibody targeting the first extracellular loop of PANX1 (Molica, et al. 2019) (Fig. 2a). In addition, a strong fluorescent signal for PANX1 (in green) was found at the plasma membrane and in the cytosol of EA.hy926 cells by immunostaining (Fig. 2b). The immunofluorescent signal was not detected in H9c2 cells (Fig. 2c) or in the negative controls (omission of primary antibody; Fig. 2b, right panel). As shown in Fig. 2d–k, the seven generated nanobodies, used at 100 nM (blue) and 300 nM (orange), show different binding affinity to the target protein in non-fixated endothelial cells. Nb1 (Fig. 2d), Nb3 (Fig. 2e), Nb6 (Fig. 2f), and Nb20 (Fig. 2i) display a concentration-dependent binding profile. Nb9 (Fig. 2g) shows a strong binding at both concentrations, indicating a high affinity for PANX1 in EA.hy926 cells. In contrast, Nb16 (Fig. 2h) exhibits a low binding affinity at the higher concentration and Nb30 (Fig. 2j) as well as the control nanobody NbR3b23 (Fig. 2k) did not bind at both concentrations tested. To determine the *K*_*d*_ for Nb1 and Nb9 in EA.hy926 cells, binding values were measured for a range of concentrations varying from 0 to 600 nM, and binding capacity curves were plotted. These results proved the higher binding affinity of Nb9 (*K*_*d*_ = 1.9 ± 1.9 nM) as compared to Nb1 (*K*_*d*_ = 387.9 ± 108.7 nM). We further confirmed the absence of Panx1 in the cardiomyocyte-like cell line at the mRNA level. Indeed, Panx1 mRNA was not detected by quantitative PCR analysis in H9c2 cells while it was readily detected in rat kidney and liver samples (Fig. 2l). The specificity of nanobody binding was demonstrated by the absence of a fluorescent signal when each of the nanobodies was used on Panx1-deficient H9c2 cells (Fig. 2m–s). Altogether, these results show that six out of seven nanobodies recognize PANX1 in endothelial cells, albeit with a different affinity. Importantly, none of the seven nanobodies exhibited non-specific binding to cardiomyocyte-like cells deficient for Panx1.

### Anti-PANX1 nanobodies do not exhibit cytotoxic effects *in vitro*

**Fig. 3:**
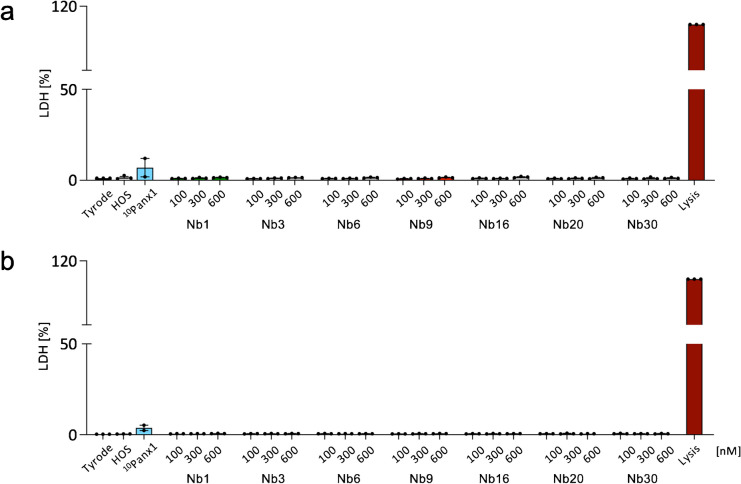
Nanobodies show no cytotoxicity in EA.hy926 and H9c2 cells. Potential cytotoxic effects were investigated by LDH release on both **a** EA.hy926 cells and **b** H9c2 cells by measuring LDH in the supernatant of these cells after 30 min of incubation with^10^Panx (400 μM) or the various nanobodies at 100 nM, 300 nM, or 600 nM. LDH in supernatant resulting from cell lysis with Triton X-100 (lysis; dark red) was used as a reference. *N* = 3

To investigate potential cytotoxic effects, we measured LDH levels in the supernatants of EA.hy926 and H9c2 cells after 30 min of treatment with each of the nanobodies at 100 nM, 300 nM, and at the double concentration of 600 nM. Figure 3 shows that virtually no LDH release was observed in the supernatant upon treatment with each of the seven nanobodies at all concentrations in either EA.hy926 cells (range 0.6 to 1.4%) or H9c2 cells (range 0.4 to 0.8%). Treatment with 400 μM ^10^Panx peptide also induced minimal LDH release (range 1.9 to 12.0%). Of note, cell lysis with the detergent Triton X-100 (2%) resulted in large amounts of LDH in the supernatants of EA.hy926 cells or H9c2 cells, further illustrating that virtually, no cell death was observed after 30 min of nanobody treatment.

### Nanobodies selectively block ATP release through Panx1 channels in endothelial cells

ATP release from endothelial cells plays an important role in cardiovascular inflammatory pathologies including cardiac I/R (Koval, et al. 2021, Tournier, 2022). As several of the generated nanobodies specifically recognized Panx1, the ability of nanobodies to block ATP release through PANX1 channels was assessed in EA.hy926 cells. PANX1 channel opening was induced in a receptor-independent manner using a hypo-osmotic shock (HOS) using previously established methods (Lamouroux, et al. 2023). ATP release from control EA.hy926 cells was 43.1 nM, which increased to 110.2 nM under HOS conditions. PANX1 channel blocking with the well-known ^10^Panx peptide (400 μM) tended to decrease HOS-induced ATP release from EA.hy926 cells and served as positive control (Table 1). We tested the seven nanobodies for their ATP release inhibitory capacity at 100 nM. Nb1, Nb3, Nb9, Nb16, and Nb20 were able to inhibit ATP release through PANX1 channels by 35% (Nb3) to > 60% (Nb20) in EA.hy926 cells at this low concentration (Table 1). To verify the specificity of PANX1 channel inhibition, we performed the same experiment using Panx1-deficient H9c2 cells. Of note, H9c2 cells exhibited a low level of ATP release upon HOS (40.9 nM), which was only one-third of the ATP release observed from a similar number of EA.hy926 cells and most likely represents ATP release through other membrane channels sensitive for HOS such as aquaporins (MacAulay 2021). Treatment with 400 μM ^10^Panx had no effect on ATP release from H9c2 cells. Likewise, treatment with 100 nM of each of the nanobodies did not significantly alter ATP release from H9c2 cells (Table 1), which underlines the specificity of their inhibitory action on PANX1 channels in EA.hy926 cells.

**Table 1:** Nanobodies inhibit PANX1 channel-mediated ATP release in a specific manner

Compound	Cell type	ATP release (% of HOS)	*P* value
^10^Panx	EA.hy926	62.5 ± 36.1	n.s
	H9c2	110.1 ± 19.2	n.s
Nb1	EA.hy926	56.8 ± 6.1	0.019
	H9c2	95.2 ± 17.8	n.s
Nb3	EA.hy926	64.9 ± 4.3	0.014
	H9c2	96.2 ± 19.0	n.s
Nb6	EA.hy926	117.7 ± 59.4	n.s
	H9c2	86.4 ± 15.7	n.s
Nb9	EA.hy926	52.6 ± 6.5	0.018
	H9c2	80.5 ± 14.2	n.s
Nb16	EA.hy926	55.6 ± 7.8	0.029
	H9c2	107.5 ± 28.1	n.s
Nb20	EA.hy926	37.2 ± 1.8	< 0.001
	H9c2	123.1 ± 31.4	n.s
Nb30	EA.hy926	67.8 ± 23.7	n.s
	H9c2	270.0 ± 181.3	n.s

ATP release was measured in endothelial EA.hy926 cells and cardiomyocyte-like H9c2 cells after pre-incubation with ^10^Panx (400 μM) or the nanobody (Nb) panel (100 nM each). Receptor-independent PANX1 channel opening was induced by a hypo-osmotic shock. All measurements were performed in hypo-osmotic shock buffer and expressed as % of control. (*N* = 3; two-tailed Student’s *t* test).

In summary, we demonstrated in in vitro experiments that the anti-PANX1 nanobodies specifically recognize and target native PANX1 channels in endothelial cells. We observed that the nanobodies varied in their affinity as well as in their PANX1 channel-blocking potential; however, none of the nanobodies were toxic for endothelial and cardiomyocyte-like cells. Together, these observations allow for the selection of two promising nanobodies based on the following criteria: (1) significant inhibition of ATP release in EA.hy926 cells > 40% (at 100 nM), (2) small positive or negative effects on ATP release from H9c2 cells (< 20% at 100 nM), (3) no cytotoxicity in EA.hy926 cells (at all concentrations used), and (4) different affinities. Nb1 and Nb9 were selected for further studies in the context of cardiac I/R.

### The anti-PANX1 nanobodies Nb1 and Nb9 decrease neutrophil adhesion to endothelial monolayers

**Fig. 4:**
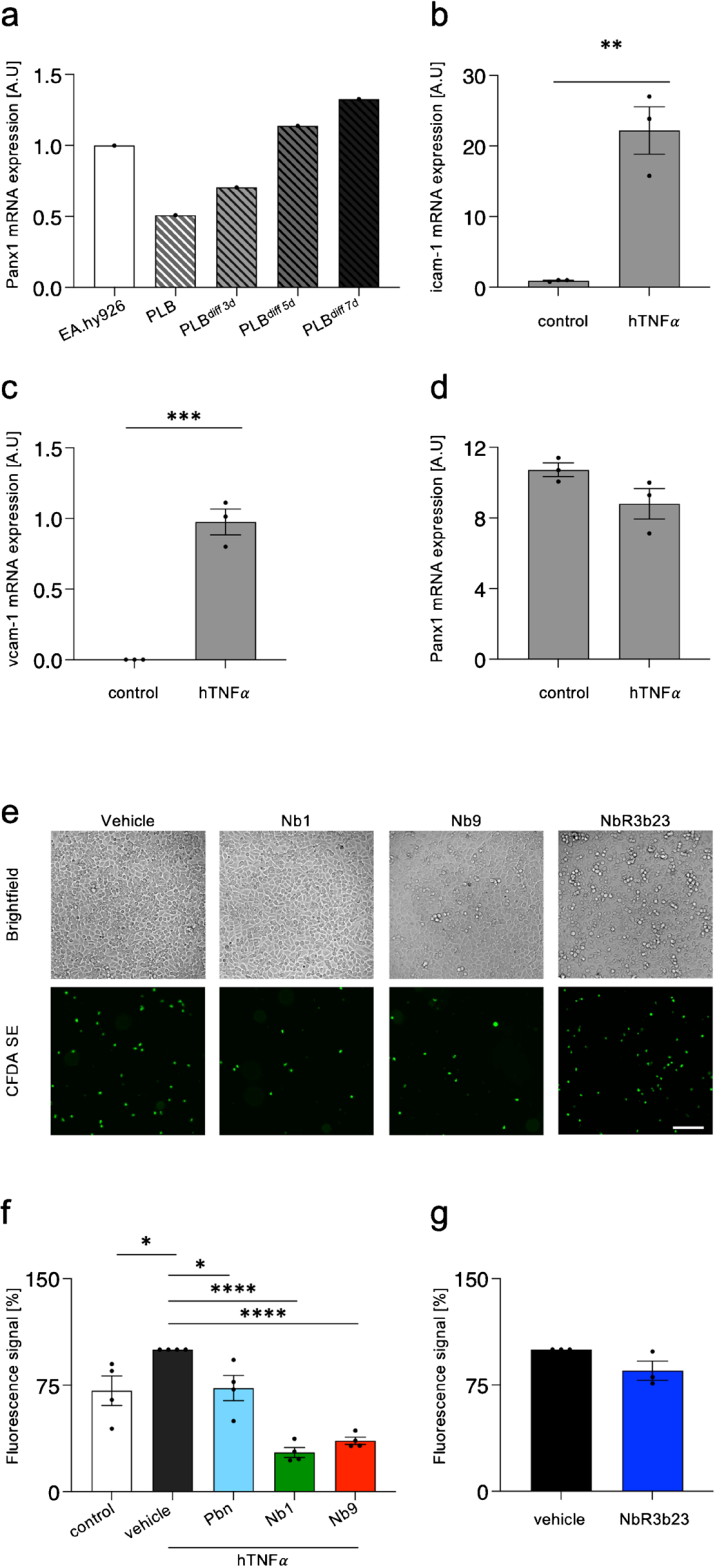
Nb1 and Nb9 decrease neutrophil adhesion to an activated endothelium. **a** Quantitative RT-PCR analysis showing the expression levels of Panx1 mRNA in human PLB-985 myeloid leukemia cells upon 3-, 5-, or 7-day DMSO-induced differentiation into neutrophils. EA.hy926 cells were used as a positive control. *N* = 1. **b**–**d** Quantitative RT-PCR analysis demonstrating the expression of icam-1 (**b**), vcam-1 (**c**), and Panx1 (**d**) mRNA in control EA.hy926 cells and after activation with 10 ng/mL hTNFα. *N* = 3 **P ≤ 0.01, ***P ≤ 0.001 (two-tailed Student’s *t* test). **e** Representative photographs of adhered CFDA-SE-stained PLB-985 cells onto monolayers of activated EA.hy926 cells under control conditions (vehicle) or after incubation of Nb1, Nb9, or NbR3b23 (100 nM). Brightfield photograph (top panels), fluorescence photograph (bottom panels). Scale bar = 100 μm. **f**–**g** Quantification of adhered PLB-985 cells onto monolayers of control and activated EA.hy926 cells incubated with vehicle (0.9% NaCl), 2.5 mM probenecid, 100 nM Nb1, Nb9, or NbR3b23. *N* = 3–4 *P ≤ 0.05, ****P ≤ 0.0001 *vs.* vehicle (one-way ANOVA)

Upon reperfusion, an exaggerated inflammatory response with the associated production of reactive oxygen species (ROS) worsens the cardiac damage. Circulating leukocytes adhere to activated endothelium via adhesion molecules (VCAM-1, ICAM-1), which are induced in response to TNFα. This cytokine is also known to induce the release of ATP through Panx1 channels in venous endothelial cells, which further augments leukocyte recruitment (Lohman, et al. 2015). To investigate the capacity of Nb1 and Nb9 to inhibit neutrophil adhesion to endothelial cells, we used the human myeloid leukemia cell line PLB-985 and differentiated them 72 h into neutrophils by the addition of 1.25% DMSO, as described previously (Ashkenazi and Marks 2009; Seredenina, et al. 2015; Tucker, et al. 1987). As shown in Fig. 4a, Panx1 expression was verified in PLB-985 cells upon different times of exposure to 1.25% DMSO, and Panx1 expression was present in PLB-985 after 72 h of differentiation to neutrophils. Next, we activated EA.hy926 cells with human TNFα for 24 h and demonstrated that the mRNA expression of icam-1 (Fig. 4b) and vcam-1 (Fig. 4c) is increased under these conditions without impacting the level of Panx1 mRNA (Fig. 4d). To investigate the anti-inflammatory potential of the nanobodies, we fluorescently labeled differentiated PLB-985 neutrophils and allowed them to adhere for 3 h onto a monolayer of TNFα-stimulated EA.hy926 cells in the presence of 100 nM Nb1 or Nb9 and 2.5 mM of the PANX1 channel blocker probenecid (positive control) or vehicle (negative control). Importantly, the number of adherent neutrophils is lower on non-activated EA.hy926 cells than on (vehicle control) endothelial cells activated with TNFα. Probenecid exerted a mild (27%) inhibitory effect on neutrophil adhesion (Fig. 4f). In contrast, both Nb1 and Nb9 reduced neutrophil adhesion by more than 64% compared to the vehicle condition (Fig. 4e, f). The control nanobody NbR3b23 had no effect on neutrophil adhesion (Fig. 4e, g). These results show that Nb1 and Nb9 can not only bind to PANX1 channels and inhibit their ATP release but that this results in reduced neutrophil adhesion to endothelial cells under inflammatory conditions in vitro.

### Administration of the anti-PANX1 nanobody Nb1 does not affect physiological cardiac function *ex vivo*

**Fig. 5:**
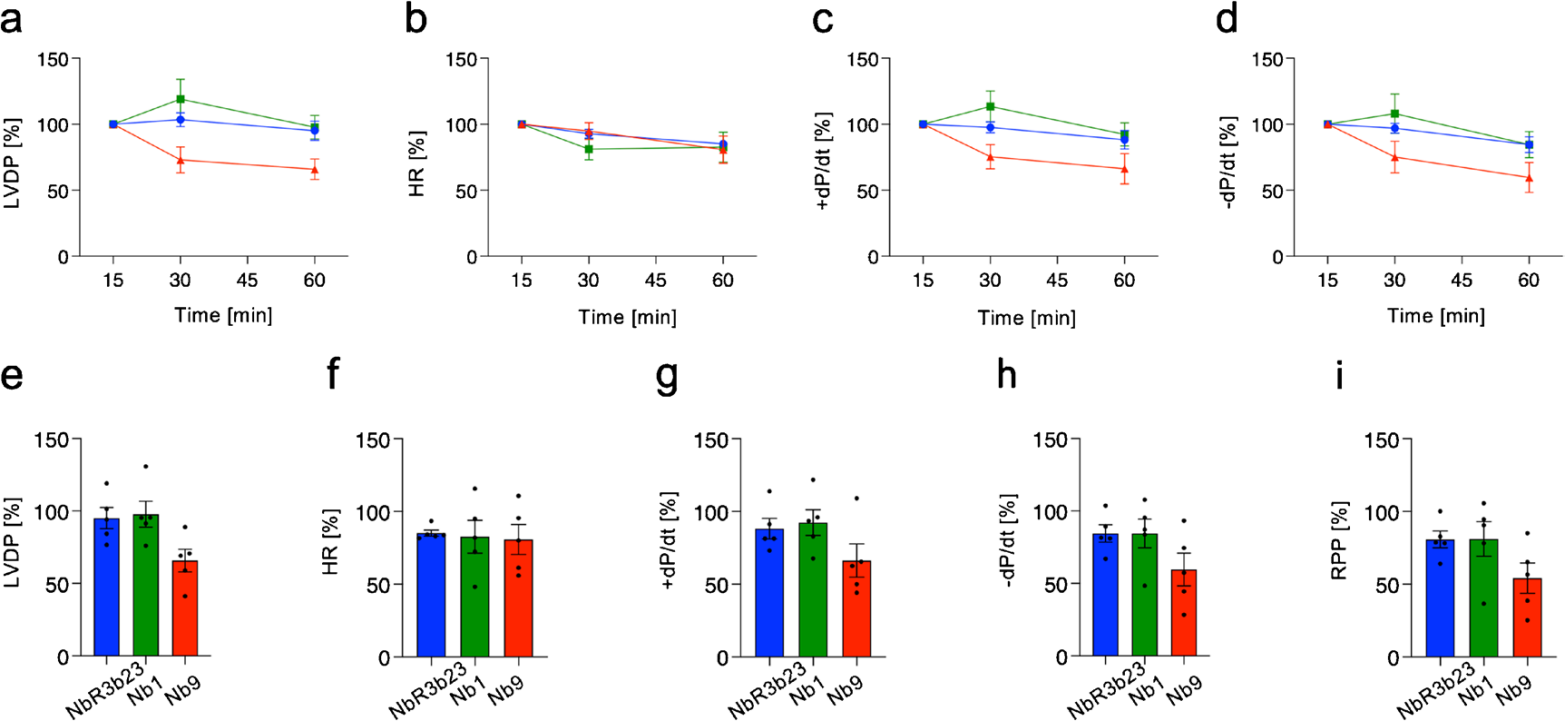
Injection of Nb1 does not impair ex vivo cardiac function. Langendorff-perfused hearts of WT mice were stabilized for 15 min after which Nb1 (green), Nb9 (red), or control NbR3b23 (blue) nanobodies were added to the perfusate for 5 min at a final concentration of 100 nM. Left ventricular function was subsequently measured for 40 min. **a**–**d** Graphs illustrating **a** left ventricular developed pressure (LVDP), **b** heart rate (HR), **c** contractility (+ dP/dt), and **d** relaxation (− dP/dt) at various time points. Nb1 and NbR3b23 nanobodies do not affect left ventricular function, while Nb9 tended to decrease LVDP, + dP/dt, and − dP/dt over time. *N* = 5. **e**–**i** After 40 min of perfusion, no significant differences were recorded in the Nb1- or Nb9-treated group compared to the control group with respect to **e** LVDP, **f** HR, **g** + dP/dt, **h** − dP/dt, and **i** rate pressure product (RPP). *N* = 5 (one-way ANOVA)

Langendorff perfused hearts permit detailed investigation of left ventricular function after exposure to nanobodies, allowing for the evaluation of their direct impact on the heart without the influence of systemic effects. We perfused the hearts of wild-type (WT) mice for 15 min to stabilize their function. At the end of the stabilization period, the left ventricular developed pressure ((LVDP) was approx. 50 mmHg, the heart rate (HR) around 260 bpm, and the contractility (+ dP/dt) and relaxation (− dP/dt) approx. 1250 and − 850 mmHg/s, respectively. Next, we administered Nb1, Nb9, or a control nanobody (NbR3b23 (Lemaire, et al. 2014)) and continued to record left ventricular function for the next 40 min. As shown in Fig. 5, Nb1 (in green) and the control nanobody (in blue) did not affect the left ventricular developed pressure (LVDP; Fig. 5a, e), heart rate (HR; Fig. 5b, f), contractility (+ dP/dt; Fig. 5c, g), or relaxation (-dP/dt; Fig. 5d, h) during this period. However, the administration of Nb9 (in red) tended to negatively impact on LVDP, left ventricle contractility, and relaxation. Finally, we calculated the rate pressure product (RPP), as an indirect measure of cardiac effort, and observed that Nb1 administration had no impact on this physiological parameter whereas RPP tended to deteriorate in the presence of Nb9 (Fig. 5i). Although no toxicity was observed in in vitro experiments, these results suggest a potential cardiotoxic effect of Nb9. Importantly, Nb1 and the control nanobody did not exhibit any negative effect on cardiac function.

### Anti-PANX1 nanobody Nb1 improves the survival of mice after *in vivo* cardiac ischemia/reperfusion

**Fig. 6:**
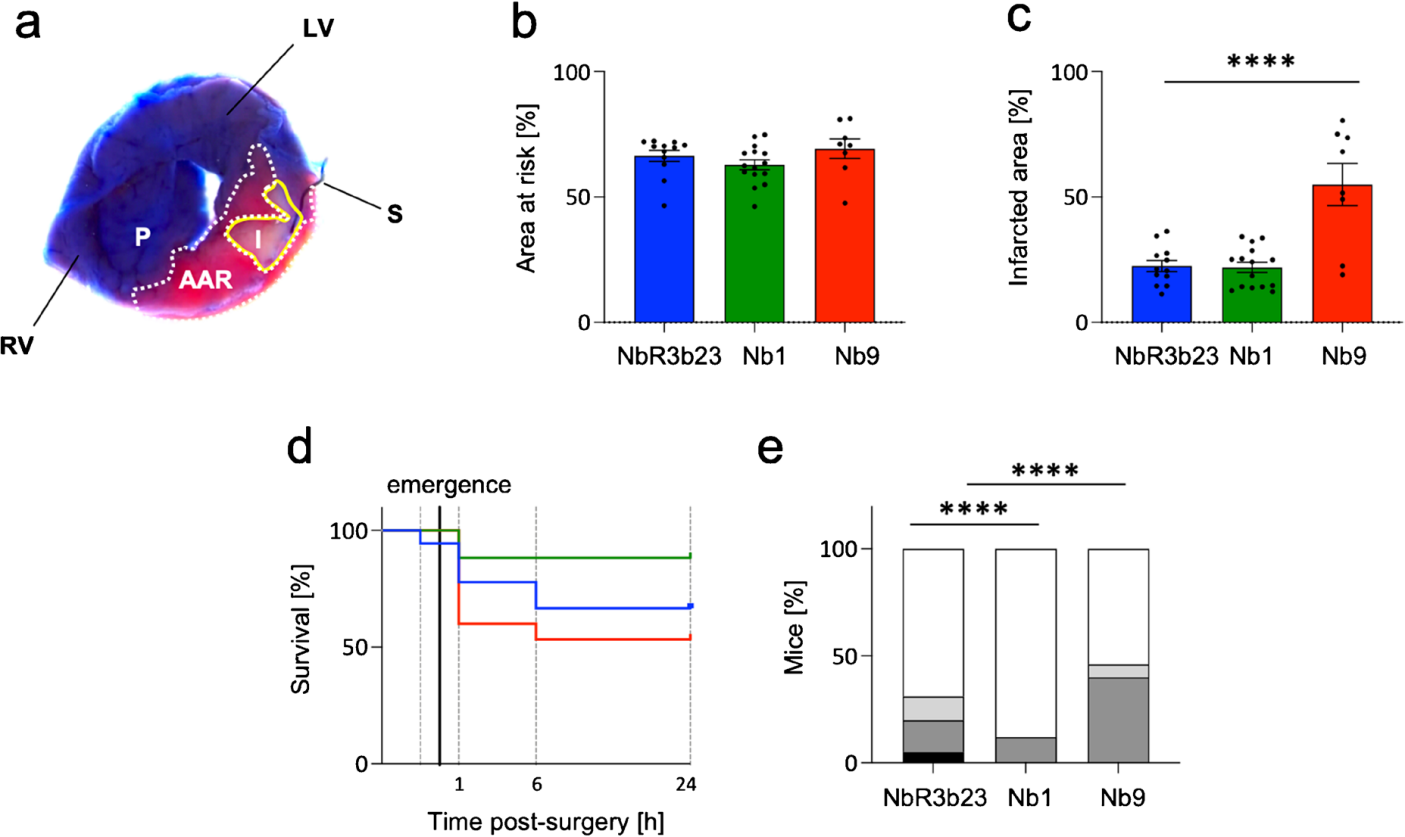
Treatment with Nb1 improves the survival of mice after cardiac ischemia/reperfusion. Mice were exposed *in vivo* to 30 min coronary artery (LAD) occlusion followed by 24-h reperfusion. Nb1, Nb9, or NbRb23 (at 20 mg/kg body weight) was administrated by tail vein injection 5 min before reperfusion. **a** The area at risk (AAR) and infarcted area (IA) were determined based on Evans blue and TTC staining, respectively. P, Evans blue positive perfused area; RV, right ventricle; LV, left ventricle; S, suture thread. **b** The AAR was similar between mice treated with control NbR3b23 (blue; *N* = 12), Nb1 (green; *N* = 15) or Nb9 (red; *N* = 8). (one-way ANOVA) **c** The IA was not different between mice treated with NbR3b23 or Nb1; however, injection of Nb9 significantly increased the infarct size. ****P ≤ 0.0001 *vs.* NbR3b23 (one-way ANOVA). **d**, **e** Nb1 improves the post-surgery survival rate. **d** Kaplan–Meier curves show the survival of mice after I/R and treatment with NbR3b23 (blue; *N* = 18), Nb1 (green; *N* = 17), or Nb9 (red; *N* = 16). Mice were divided into groups according to their time of death (X axis): (1) from the administration of nanobodies until emergence from anesthesia; (2) within the first hour from emergence from anesthesia; (3) from 1 to 6 h post-I/R; and (4) from 6 to 24 h post-I/R. NbRb23-treated mice showed expected death rate. A better survival of mice after Nb1 treatment and a decreased survival after Nb9 injection compared to the control NbR3b23 was found. **e** The percent of mice dying in the afore-described time periods (as compared to the total number of mice in each group). None of the Nb1- or Nb9-treated mice died before the emergence from anesthesia, while the mortality rate in this category in the control group was 5% (black). The percentage of mice that died within the first hour after emergence from anesthesia (dark grey) was 15%, 12%, and 40% in the NbR3b23, Nb1, and Nb9 injected groups, respectively. As such, 11% of mice injected with NbR3b23 and 6% of mice injected with Nb9 died between the 1st and 6th h post-emergence (light grey). Finally, 69% of control mice survived the 24 h reperfusion period and were sacrificed *per protocol* (white), which corresponds to 88% in Nb1 and 54% of Nb9 group. ****P ≤ 0.0001 *vs.* NbR3b23 (Fisher’s exact test)

To study the cardioprotective potential of Nb1 and Nb9 *in vivo*, we subjected WT mice to 30 min of ischemia by occluding the left anterior descending coronary artery (LAD) followed by 24 h of reperfusion. Nb1, Nb9, or control NbR3b23 was intravenously administrated 5 min prior to reperfusion at a concentration of 20 mg/kg body weight. The area at risk (AAR) and the infarcted area (IA) were determined using well-established protocols with Evans blue and TTC staining (Lecour, et al., 2021). As expected, the AAR was similar for each group (Fig. 6a), underlining the reproducibility of the experimental procedure. Whereas Nb1 did not affect the IA compared to the control nanobody, treatment with Nb9 augmented the infarct size (Fig. 6b). We also established a Kaplan–Meier curve to compare the survival of mice subjected to cardiac I/R with each nanobody (Fig. 6c). The survival rate of mice treated with control nanobody was in the range that is expected for this invasive procedure. Remarkably, we observed that a significantly higher number of mice survived during the 24-h period after cardiac I/R in the Nb1-injected group (88%) (Fig. 6d). In fact, Nb1-treated mice only died within the first hour of the post-surgical period and seemed protected thereafter. As expected, the total procedural death rate in the NbRb23 group was around 30% and observed before emergence from anesthesia, during the first hour and in the 1-to-6-h post-surgery period (Fig. 6d). The survival of Nb9-treated mice was significantly lower than both the control and Nb1-treated groups, which was mostly due to a decreased survival rate in the first-hour post-surgery (Fig. 6d).

Given the preservation of physiological cardiac function together with improved survival of mice after *in vivo* I/R, Nb1 appears as a promising therapeutic tool to confer cardioprotection by blocking PANX1 channels.

## Discussion

In recent years, it has been demonstrated using various mouse disease models that genetic deletion or pharmacological blocking of PANX1 channels induces protection against I/R injury in several organs including the heart (Good, et al. 2021; Koval, et al. 2021; Rusiecka, et al. 2023, Tournier, 2022). The currently used pharmacological Panx1 channel inhibitors, such as carbenoxolone, probenecid, and spironolactone, were found to act in a non-selective manner as they also target connexin channels, organic anion transporters, or mineralocorticoid receptors (Davidson, et al. 1986; Perwitasari, et al. 2013; Rusiecka, et al. 2022). The introduction of synthetic peptides targeting specific sequences has overcome this hurdle. A 10-amino acid–long peptide derived from EL1, called ^10^Panx, has been shown to block PANX1 channels and to possess beneficial anti-inflammatory effects both in vitro and in vivo (Maes, et al. 2017; Pelegrin and Surprenant 2006). Another peptide, TAT-Panx1_308_, contains a TAT sequence that allows it to enter the cell where it specifically recognizes and blocks phosphorylation of PANX1-Tyr308, thereby preventing the activation of PANX1 channels by Src Family Kinases (SFKs) during anoxia (Weilinger, et al. 2016). Such linear peptides possess however limited plasma stability, which restricts their in vivo use, and current studies focus on improving their proteolytic stability (Tournier, 2022). In 2019, we developed a mini-antibody specifically targeting PANX1-EL1, which was able to reduce hemostasis and thrombosis *in vivo* (Molica, et al. 2019). The variable domain of the camelids’ heavy-chain antibodies, known as nanobodies, offers now new technological possibilities. Because of their small size, high target affinity, and deep tissue penetration and their ability to target clefts that are inaccessible to monoclonal antibodies, nanobodies have a great translational potential (Danquah, et al., 2016, McMahon, et al. 2020; Muyldermans 2021). Nanobodies targeting PANX1 have been recently introduced (Van Campenhout et al. 2023). In the present study, we demonstrated by FACS and ATP release experiments (Fig. 2; Table 1) that Nb1 and Nb9 have different affinity (*K*_*d*_ = 387.9 nM for Nb1 and 1.9 nM for Nb9) in human endothelial cells but exhibit both high target selectivity similar to previous observations in DUBCA cells overexpressing mouse or human PANX1 (Van Campenhout et al. 2023). Importantly, no in vitro toxicity was observed in endothelial and cardiomyocyte-like cells (Fig. 3), paving the way for in vitro adhesion assays, cardiac function studies in isolated hearts, and finally *in vivo* cardiac I/R experiments.

We showed in this study that both Nb1 and Nb9 decrease neutrophil adhesion to an activated endothelial cell monolayer (Fig. 4). These effects were even stronger than the ones of probenecid, being the positive control, underlining the high efficiency of nanobodies in reducing neutrophil adhesion, a first and obligatory step in the inflammatory cascade. During the initial phase, neutrophils roll along the vascular endothelium. Firm adhesion is mediated by the increased expression of adhesion molecules, like VCAM-1 and ICAM-1, at the surface of endothelial cells upon activation by cytokines such as TNFα. TNFα is also known to activate endothelial PANX1 channels via Src-dependent phosphorylation (Lohman, et al. 2015). ATP released by endothelial PANX1 channels serves as a chemoattractant for neutrophils, enhancing their recruitment to the injured area where they are an important source of ROS, which augments the cardiac injury upon reperfusion (Heusch 2020). Both Nb1 and Nb9 decreased neutrophil adhesion to activated endothelial cells (Fig. 4d), thus providing further in vitro evidence that molecular exchange through PANX1 channels contributes to the inflammatory response. Moreover, these results place Nb1 and Nb9 among the nanobodies with anti-inflammatory potential (Bertheloot, et al. 2022; Rissiek, et al. 2014; Van Bockstaele, et al. 2009). Indeed, nanobodies against pro-inflammatory cytokines have been shown to attenuate their effects and neutralize inflammation, such as Ozoralizumab against TNFα (Rissiek, et al. 2014). Moreover, the nanobodies against the inflammasome adaptor protein ASC were recently shown to disassemble the post-pyroptotic inflammasome and to neutralize the effect of gout (Bertheloot, et al. 2022).

To further investigate the potential adverse effects of Nb1 and Nb9 on cardiac function, we performed ex vivo experiments on isolated Langendorff-perfused mouse hearts. Nb1 and the control NbR3b23 did not affect LVDP, contractility, relaxation, HR, and RPP during the 40 min following their application. In contrast, these parameters tended to decrease in Nb9-treated hearts, suggesting that Nb9 may negatively affect cardiac function. Notably, Nb9 had no influence on HR, showing that the putative toxic effects of Nb9 are primarily related to contractile force and relaxation rather than general cardiotoxic effects. A limitation of our ex vivo experiments is that the nanobodies could only be used at a single concentration. One might thus consider a more effective PANX1 channel blockade by Nb9 due to its higher affinity than Nb1 as a potential alternative explanation for the decreased cardiac function. However, the fact that physiological cardiac function in Langendorff-perfused WT and *Panx1*^*−/−*^ hearts are not different (Rusiecka, et al. 2023) makes this possibility unlikely. The Langendorff isolated mouse heart model is widely employed to study myocardial function in response to injury or pharmacological interventions. Nonetheless, important methodological variations exist, for instance in the composition of the perfusion fluid, strain, age and sex of mice, constant pressure or constant flow perfusion, and the use of pacing (Liao, et al. 2012; Louradour, et al. 2023; Noly, et al. 2022; Reichelt, et al. 2009). These variations may affect the observed values for LVDP, contractility, relaxation, and HR, as well as the sensitivity to observe changes in cardiac function, which represents a limitation of this ex vivo method. The LVDP, contractility, relaxation, and HR measured in the present study were of comparable magnitude to earlier studies from our and other laboratories, in which differences in cardiac function have been detected (Louradour, et al. 2023; Morel, et al. 2014, 2016; Rusiecka, et al. 2023). Although it cannot be completely ruled out, we consider it unlikely that the absence of cardiotoxic effects of Nb1 are due to a too low sensitivity for detecting effects of nanobodies on cardiac function.

We studied the potential cardioprotective effects of the nanobodies following cardiac I/R *in vivo*. AAR is an established indicator of the reproducibility of surgical technique. Notably, this parameter is comparable in each group (Fig. 6a). While treatment with Nb1 and the control NbR3b23 resulted in a similar infarct size (20–22%), mice treated with Nb9 had significantly larger infarcts, reaching an average of almost 55%. Together with the ex vivo decline in cardiac performance, this points to a potential cardiotoxic effect of Nb9. While nanobodies are often described as being less immunogenic than conventional antibodies, toxic effects following *in vivo* administration have previously been reported but were mostly linked to the rapid renal clearance of nanobodies and their accumulation in the kidney (Hosseindokht, et al. 2021; Jovcevska and Muyldermans 2020; Van Audenhove and Gettemans 2016). It should however be noted that the infarct size was measured only in mice that survived the 24-h recovery period, and thus likely represents an underestimation for the groups with higher mortality when compared to groups with lower mortality. An eventual beneficial effect on the infarct size of Nb1 might thus have been masked by the differences in mortality rate between the groups treated with Nb1 and the control nanobody. In an earlier study, *Panx1*^*−/−*^ mice showed a decreased sensitivity to cardiac I/R injury, as compared to WT mice, at 24 h after reperfusion, resulting in smaller infarcts and improved recovery of left ventricular function. This cardioprotective effect of Panx1 deletion involved cardiac mitochondria. In general, the cell plasma membrane is a barrier for nanobodies, and adapted delivery methods might be needed to reach intracellular targets such as mitochondria (de Beer and Giepmans 2020). Whether the PANX1-targeting nanobodies can cross the plasma membrane is presently unknown.

Strikingly, mice treated with Nb1 had much improved survival following *in vivo* cardiac I/R. As expected, we observed about 30% of mortality 24 h after surgery in the NbR3b23-treated group. This overall mortality was reduced by 23% in the Nb1-treated group. It is probable that Nb1 induces a protective mechanism that does not directly reduce the inflammatory response. Indeed, our recent work could not reveal a difference in neutrophil recruitment at 24 h after cardiac I/R between control and *Panx1*^*−/−*^ mice (Rusiecka, et al. 2023). Moreover, neutrophil-specific or endothelial-specific deletion of Panx1 did also not affect infarct size at 24 h following cardiac I/R (Good, et al. 2021; Rusiecka, et al. 2023). Instead, Nb1 may activate an earlier window of protection resulting in improved recovery of cardiomyocytes. The notion of “first” and “second” window of protection (SWOP) has been introduced in the context of ischemic preconditioning (IPC) (Hausenloy and Yellon 2010). Indeed, brief cycles of ischemia followed by reperfusion prior to the prolonged ischemia trigger a range of cellular responses at various time intervals post-reperfusion (Hausenloy and Yellon 2010; Rossello and Yellon 2018). The first phase of protection induces activation of the reperfusion injury salvage kinase (RISK) and the survivor activating factor enhancement (SAFE) cascades within cardiomyocytes that target mitochondria and manifests an immediate response improving cell survival (Rossello and Yellon 2018). The second phase occurs later on, after 12–24 h post-reperfusion exerting beneficial effects on coronary endothelial cell function through manipulation of ROS signaling cascades (Hausenloy and Yellon 2010) and diminished neutrophil adhesion (Hausenloy and Yellon 2010; Laude, et al. 2002). We have recently shown that Panx1 deletion, by changing mitochondrial respiration and ATP handling, renders cardiomyocytes less sensitive to ischemic injury and improves the myocardial recovery post-I/R (Rusiecka, et al. 2023). Although it is unlikely that Nb1 reached mitochondrial PANX1 channels, one can imagine that Nb1-mediated inhibition of PANX1 channels at the plasma membrane may induce molecular responses in cardiomyocytes that modulate mitochondrial sensitivity to reperfusion leading to the preservation of mitochondrial function and ATP synthesis. Although this hypothesis is tempting, the present study was exclusively designed to measure cardioprotective outcomes in a quantitative manner, and detailing the molecular mechanism remains the subject of future investigations. Such investigations should include, but are not limited to, the effects of Nb1 on PANX1 expression in endothelial cells as well as cardiac cells in vitro and in vivo, effects of Nb1 on cell metabolism, and electrophysiology to determine the effect of Nb1 on PANX1 channel properties in detail.

## Data Availability

Source data of results presented in this manuscript are available at Yareta, the consultation and archiv portal for Geneva’s institutions of higher learning (10.26037/yareta:yqceixq6i5dbzdj2jlte5sunmm).
